# A white paper from the FEBS Education and Training Conference: challenges, opportunities, and action plans for transforming molecular life sciences education

**DOI:** 10.1002/2211-5463.13916

**Published:** 2024-12-12

**Authors:** Ly Villo, Nino Sincic, Luciane V. Mello, Manuel Joao Costa, Didier Picard, Francesco Malatesta, Jerka Dumic, Ferhan G. Sagin

**Affiliations:** ^1^ Department of Chemistry and Biotechnology Tallinn University of Technology Estonia; ^2^ Member of FEBS Education and Training committee; ^3^ Department of Medical Biology, School of Medicine University of Zagreb Croatia; ^4^ Department of Molecular Biology Center of Excellence for Reproductive and Regenerative Medicine Zagreb Croatia; ^5^ Institute of Systems, Molecular and Integrative Biology, School of Biosciences University of Liverpool UK; ^6^ School of Medicine University of Minho Braga Portugal; ^7^ Department of Molecular and Cellular Biology University of Geneva Switzerland; ^8^ Department of Biochemical Sciences Sapienza University of Rome Italy; ^9^ Faculty of Pharmacy and Biochemistry University of Zagreb Croatia; ^10^ Department of Medical Biochemistry Ege University Medical School Izmir Türkiye

**Keywords:** active learning and engagement, call for action in molecular life sciences education, digital revolution in education, educating educators, Education and Training Conference, education in molecular life sciences

## Abstract

The inaugural FEBS Education and Training Conference (ETC) was held in Türkiye, in 2024. This first‐ever Molecular Life Sciences Education Conference in Europe was organized by the FEBS Education and Training Committee and it was a groundbreaking event that brought together educators and scientists to explore how to enhance education and training in molecular life sciences. The conference explored a wide range of critical themes, for example—digital revolution, active learning and student engagement, multidisciplinary teaching and learning, transitions and inclusivity in education, students' self‐assessment, and self‐regulated learning. The discussions and presentations underscored the pressing need for transformation in how academics and researchers approach teaching and learning. Such shift is driven by rapid technological advancements, societal shifts, and the evolving demands of the scientific landscape. This document synthesizes key insights, discussions, and recommendations from the conference and proposes actionable strategies for all stakeholders in the field—institutions, educators and students—to address current challenges in education and training in molecular life sciences.

## Graphical abstract

The first molecular life sciences education conference in Europe—FEBS Education and Training Conference—brought together educators and scientists to explore how to enhance education and training and underscored the need for transformative changes in teaching and learning. This document synthesizes key insights and recommendations from the conference and proposes strategies to address current challenges in education.

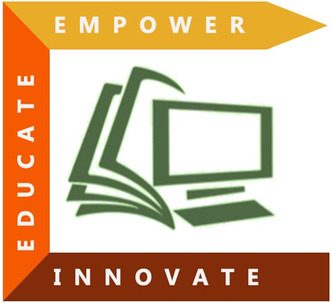

**Abbreviations**
AI: artificial intelligence
DELTA: the disciplinary excellence in teaching, learning, and assessment
EAT: equity, agency and transparency
ETC: education and training conference
OER: open educational resources
VALUE: valid assessment of learning in undergraduate education

The inaugural FEBS Education and Training Conference (ETC) was held in Antalya, Türkiye, between March 20 and 23, 2024. The event was organized by the FEBS Education and Training Committee (Conference Chair Ferhan G. Sagin—Türkiye and co‐chair Nino Sinčić—Croatia). This first‐ever Molecular Life Sciences Education Conference in Europe was a groundbreaking event that brought together educators and scientists to explore how to enhance education and training in molecular life sciences. There were 118 participants from 29 countries. The program was rich with 1 panel discussion, 5 plenary lectures, 10 invited talks, 1 satellite symposium, 8 workshops, 16 short presentations, and 17 poster presentations (Fig. 1).

**Fig. 1:**
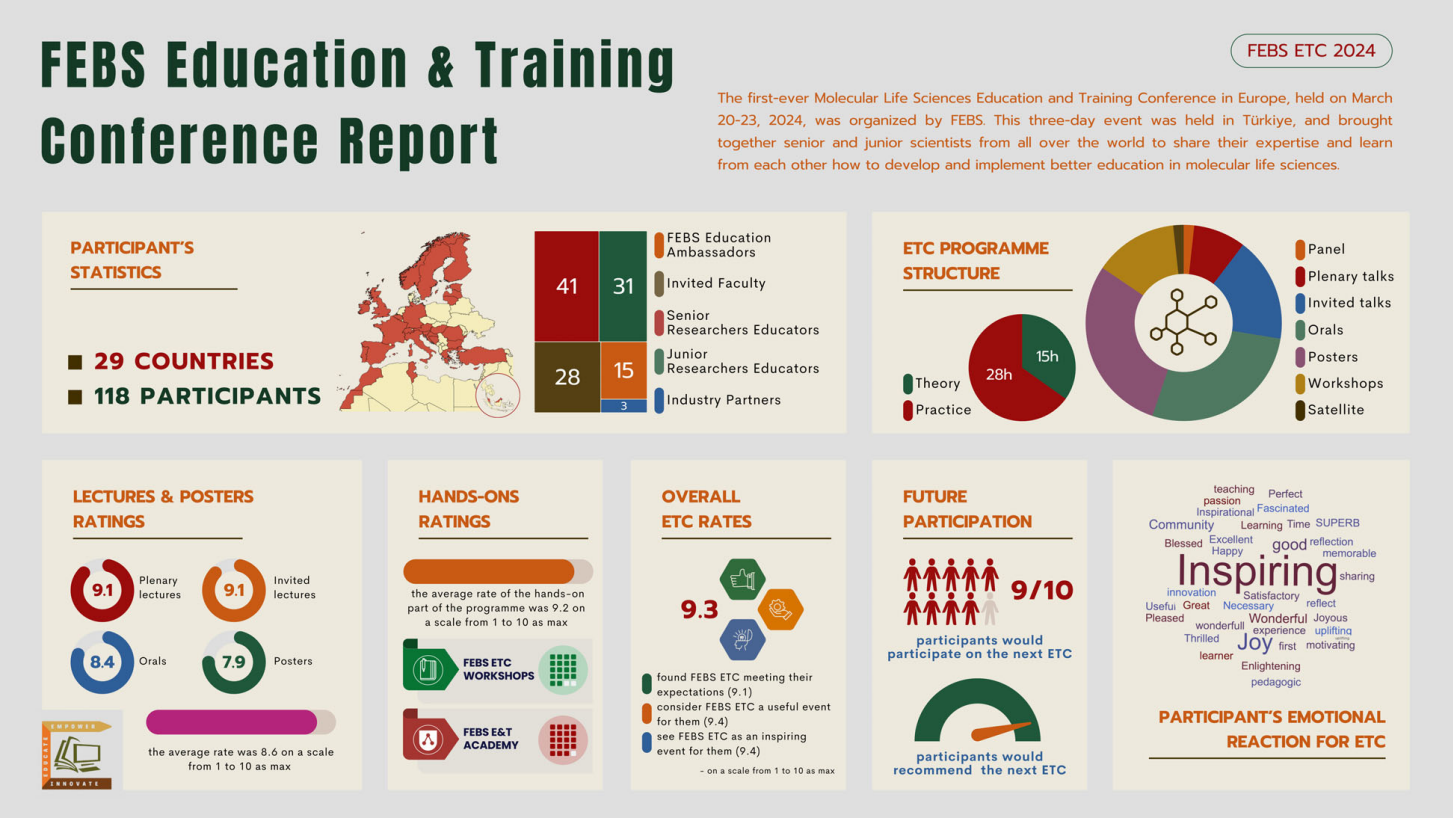
Overview of the inaugural FEBS Education & Training Conference (ETC) 2024. The infographic summarizes key statistics, including participant demographics, program structure, session ratings, and overall participant satisfaction from the first Molecular Life Sciences Education and Training Conference in Europe, held in Türkiye, March 20–23, 2024. The data highlight the conference's international reach, diverse program offerings, and positive reception among attendees.

The discussions and presentations underscored the pressing need for transformation in how academics and researchers approach teaching and learning. Such shift is driven by rapid technological advancements, societal shifts, and the evolving demands of the scientific landscape. This document synthesizes key insights, discussions, and recommendations from the conference, with the aim of raising awareness about the critical importance of education and training in molecular life sciences. It calls for actionable steps to address current challenges, while fostering innovation and collaboration to ensure a bright future for education in this field.

## Key themes and challenges

**Fig. 2:**
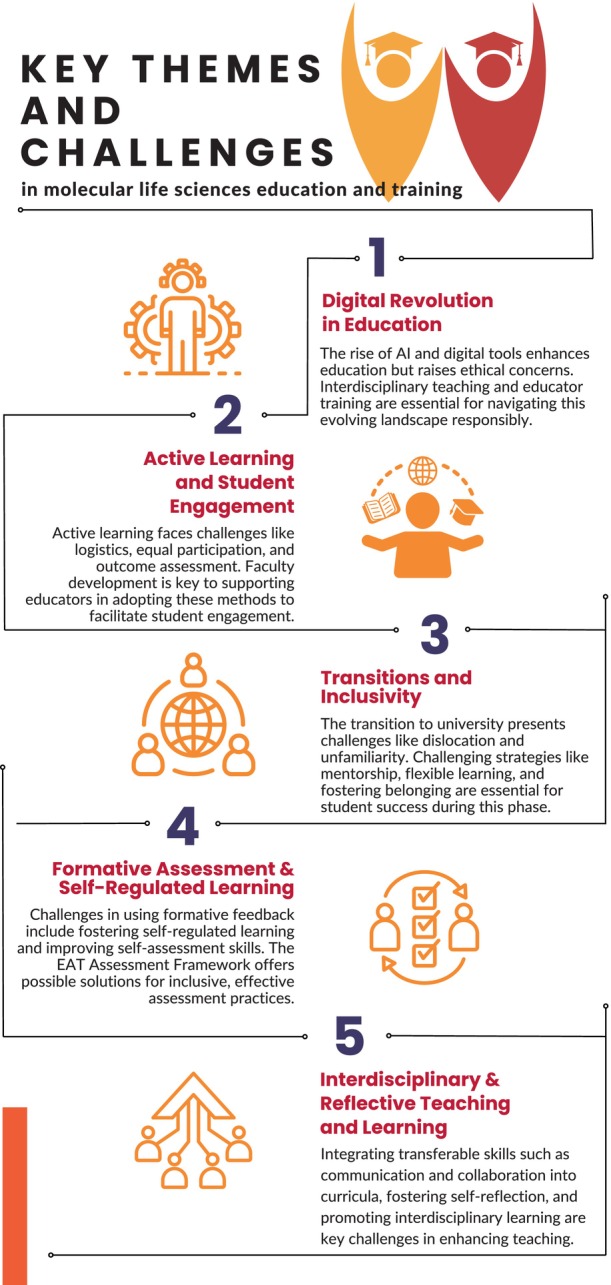
Key Themes and Challenges in Molecular Life Sciences Education and Training. This infographic illustrates the five primary areas of focus identified during the FEBS Education & Training Conference 2024: (1) Digital Revolution in Education, highlighting the transformative potential and ethical considerations of AI and digital tools; (2) Active Learning and Student Engagement, emphasizing the importance of interactive and student‐centered approaches; (3) Transitions and Inclusivity, addressing challenges in the school‐to‐university transition; (4) Formative Assessment & Self‐Regulated Learning, focusing on enhancing student self‐assessment and motivation; and (5) Interdisciplinary & Reflective Teaching and Learning, stressing the integration of transferable skills and interdisciplinary approaches in education. Each theme encapsulates key discussions and challenges in advancing molecular life sciences education.

The conference explored a wide range of critical themes each emphasizing the complex and evolving nature of modern education (Fig. 2).

Central to these discussions was the transformative potential of the digital revolution, which emerged as a recurring and dominant theme throughout the event. The integration of technology into teaching and learning practices was lauded for its potential to enhance engagement, accessibility, and efficiency. However, the ‘power and perils of AI’ were also acknowledged, prompting discussions on the ethical implications, potential biases, and the need for critical thinking in the digital age. The discussion also highlighted topics such as the digital divide and teaching on open‐source platforms while recognizing artificial intelligence (AI) biases, particularly from Eurocentric data. The conference underscored the importance of equipping educators with the skills and knowledge to navigate the complexities of the digital landscape, ensuring that technology serves as an enabler rather than a disruptor of effective learning.

The traditional teaching‐learning model, focused on facts and linear teaching, was deemed inadequate for the future. Emphasis should be on active learning, multidisciplinary teaching, authentic and experimental learning, and the cultivation of personal development traits like resilience and grit.

**Fig. 3:**
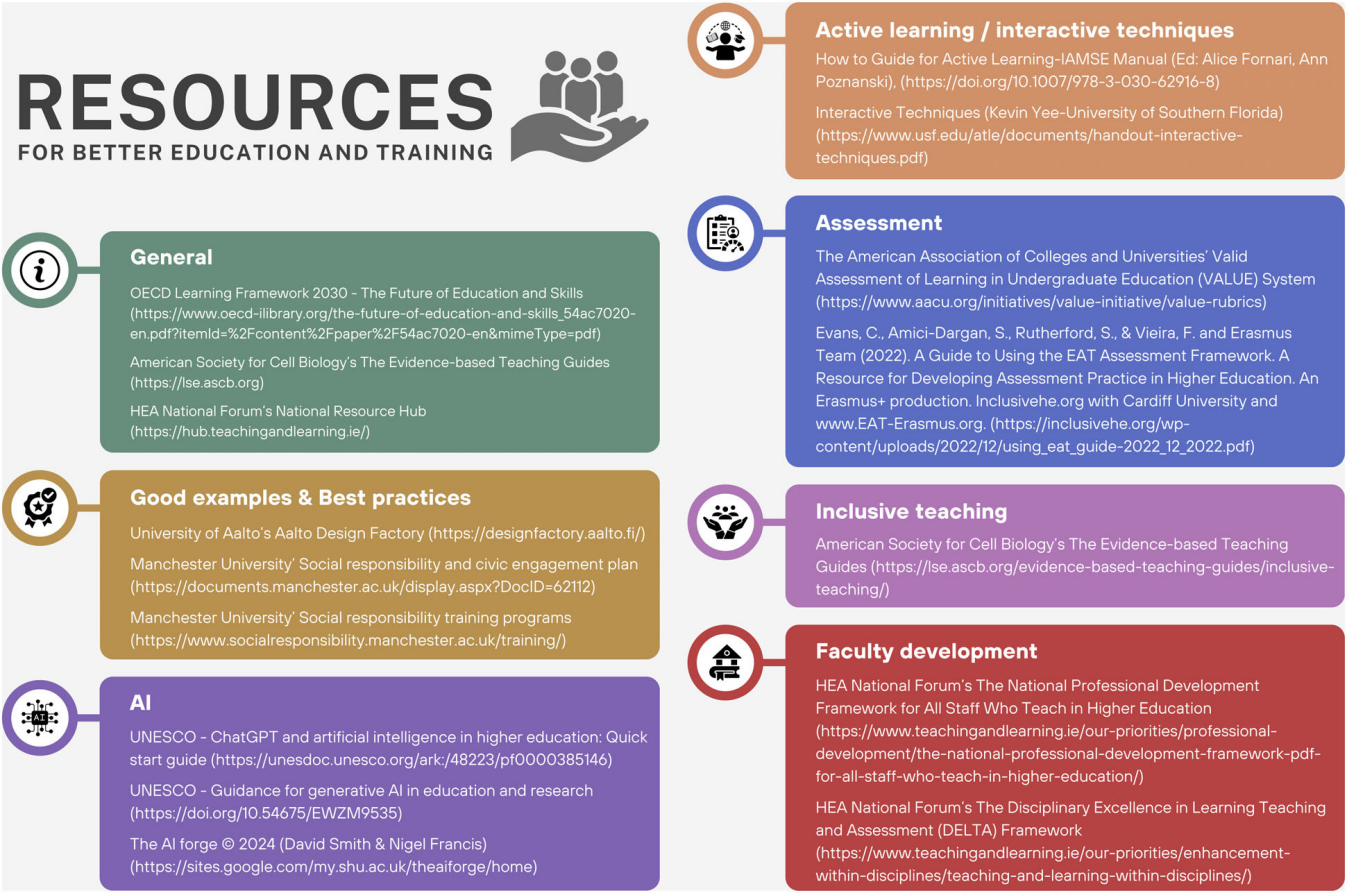
Some Resources for Better Education and Training in Molecular Life Sciences. This infographic highlights a selection of resources, best practices, and frameworks that emerged from or were suggested at the FEBS Education & Training Conference 2024. The figure presents seven categories of resources: (1) General frameworks, (2) Good examples & best practices, (3) Active learning & interactive techniques, (4) Inclusive teaching, (5) AI in education, (6) Assessment tools, and (7) Faculty development. While not exhaustive, these resources represent key areas of focus for enhancing molecular life sciences education and training, as discussed during the conference.

The future of education, as outlined in the OECD Education Framework 2030 (The Future of Education and Skills—Education 2030), emphasizes the interconnectedness of knowledge, skills, attitudes, and values. In alignment with this framework, it is also strategic to develop the assessment of noncognitive competencies as they are essential to both the individual and collective well‐being. More examples such as the innovative approach of Aalto University's Design Factory, with franchises in many countries, to solve real‐world problems are needed. Future skills, as outlined by the VALUE (Valid Assessment of Learning in Undergraduate Education) rubrics, are emphasized. Additionally, new learning opportunities, such as Manchester University's emphasis on social responsibility and making a real‐world impact, are central to this evolving educational paradigm (Fig. 3).

In today's rapidly evolving educational landscape, the human–human interaction remains crucial, necessitating significant changes in assessment and monitoring practices. The conference also addressed the profound transformation over the past decade which expanded the role of educators beyond traditional boundaries. A modern, effective teacher was proposed to be the one who challenges conventional notions, making learning as dynamic and engaging as our ever‐changing world. Today's educators must possess expanded knowledge structures that encompass social, international, and cultural dimensions. Their skill set should now include the ability to navigate disciplinary boundaries, foster digital literacy, and appreciate cultural diversity. While the fundamental role of an educator—to guide and inspire learning—remains unchallenged, there is an increased emphasis on adaptability and a commitment to evolving educational values.

To become effective change agents, educators need to move away from a ‘banking education system’, that is, an educational system that focuses solely on the acquisition of knowledge and skills. Instead, they need to embrace an educational approach that cultivates a professional identity aligned with the values of good research practice. This paradigm requires curricula that views change as a continuum and a process of continuous improvement, encouraging self‐reflection and critical thinking. Educators must prepare for the future by engaging in collaborative learning with students, peers, and society, while collectively adapting to new challenges.

The transformative power of education lies in its ability to inspire joy, foster to foster self‐criticism, and to celebrate diversity. It was agreed that only by embodying these principles, education can empower learners to become agents of change in an ever‐evolving world. The purpose of education extends beyond mere qualification; it encompasses socialization and the development of an individual professional identity. This holistic approach aims to foster student development so they can navigate complex societal challenges, to contribute meaningfully to their communities, and to continuously adapt to a changing global landscape. By focusing on these multifaceted goals, education can truly become a catalyst for personal growth and societal progress.

More details about these critical themes are given below.

### The digital revolution in education

Discussions at the conference emphasized the profound impact of technological advancements on the educational landscape. The rapid changes in higher education, particularly those resulting from the COVID‐19 pandemic and advancements in AI, were key focal points.

The integration of technology into teaching and learning practices has the ability to enhance engagement, accessibility, and efficiency. Transformative digital learning tools enable the next generation of scientists to develop an experimental mindset, build scientific skills, competencies and attitudes, and be prepared for the future workplace. Technologies support the creation of interactive, problem‐solving laboratory simulations and advanced digital worksheets that enable self‐led learning and improvement. These digital tools enable students to practice, to apply feedback, and to master the advanced skills needed for excellent scientific literacy. The conference showcased these innovative approaches to leveraging technology, from interactive laboratory simulations and digital worksheets to AI‐driven data analysis and assessment tools.

The event highlighted the importance of interdisciplinary collaboration and the integration of diverse subjects to create comprehensive and relevant curricula. The rise of AI and its impact on various fields, including drug discovery, molecular modeling, and precision medicine, necessitates a shift toward an interdisciplinary approach that equips students with the skills and knowledge to navigate the complexities of the modern scientific landscape, necessitating the fusion of diverse subjects to create comprehensive curricula.

However, the ‘power and perils of AI’ were also discussed, sparking thoughtful discussions on the ethical implications, potential biases, and the need for critical thinking in the digital age. Discussions highlighted concerns about digital footprints and the environmental impact, the importance of critical evaluation, and responsible use of technology. The need for tailored approaches in teaching and learning were emphasized ensuring that technology serves as a complement to, rather than a replacement for, traditional teaching methods.

The conference underscored the importance of equipping educators with the skills and knowledge to navigate the complexities of the digital landscape, ensuring that technology serves as an enabler rather than a disruptor of effective learning.

### Active learning and student engagement

A key theme that emerged from the conference was the critical importance of active learning, particularly in addressing the engagement challenges often faced in large classes. Presenters and participants alike emphasized that active learning is not just a trendy concept, but a crucial strategy for improving student engagement and learning, deepening understanding, and fostering critical thinking skills.

Several innovative approaches to active learning were showcased:

1. Interactive Activities: Presenters demonstrated how incorporating hands‐on, problem‐solving tasks can transform traditionally passive lectures into dynamic learning experiences. These activities ranged from short, in‐class exercises to more complex, technology‐enabled simulations.
2. Group Discussions: The power of peer‐to‐peer learning was highlighted through structured group discussions. Techniques such as think‐pair‐share and jigsaw discussions were presented as effective ways to promote collaborative learning and expose students to diverse perspectives.
3. Peer Teaching: Conference participants shared success stories of implementing peer teaching strategies, where students take turns explaining concepts to their classmates. This approach not only reinforces learning for the student–teacher but also provides relatable instruction for the listeners.
4. Technology‐Enhanced Learning: Various digital tools and platforms were discussed as means to facilitate active learning in large classes. These included real‐time polling systems, collaborative online workspaces, and gamified learning applications.
5. Flipped Classroom Model: Several presenters advocated for the flipped classroom approach, where lecture content is accessed online before class, allowing in‐person time to be dedicated to active problem‐solving and discussions.

The conference also addressed the challenges of implementing active learning strategies, particularly in large classes. Presenters discussed methods for managing logistics, ensuring equitable participation, and assessing learning outcomes in active learning environments. The importance of faculty development programs to support educators in adopting these methods was also emphasized.

As the conference concluded, there was a clear consensus that the future of education lies in moving away from passive, lecture‐based instruction toward more engaging, student‐centered approaches. The challenge now lies in scaling these practices across institutions and disciplines, ensuring that active learning becomes the norm rather than the exception in higher education.

### Transitions and inclusivity in education

The transition from high school to university is a pivotal juncture in a student's academic journey. Acknowledging this significant leap from school to university and recognizing the significant challenges faced by students during this transition are critically important. Identifying obstacles that hinder effective learning during the transition and addressing ‘dislocation, loss of belonging, and unfamiliarity’ experienced by students necessitate proactive measures to ensure a smooth and successful passage.

The conference explored strategies to empower students during this critical phase, emphasizing the importance of bridging the gap between secondary and tertiary education. Strategies to support students during this transition were discussed, including the use of technology to create flexible learning opportunities and the importance of fostering a sense of belonging, adaptability, and self‐regulated learning. Strategies such as mentorship programs, orientation initiatives, and the cultivation of a supportive learning environment were proposed to empower students during this critical phase.

### Formative assessment and self‐regulated learning

The role and importance of formative feedback in reinforcing self‐regulated learning strategies, enhancing motivation, and improving academic outcomes were emphasized. Techniques to enhance students' self‐assessment abilities and the use of feedback to drive lifelong learning were key points of discussion. The concepts of ‘self‐assessment ability’ and ‘self‐regulation promoting students’ self‐evaluation skills' were explored and the Equity, Agency and Transparency (EAT) Assessment Framework was highlighted as a research‐informed approach and tool for educators to implement effective inclusive assessment practices.

### Interdisciplinary and reflective teaching and learning (including professional development)

The cultivation of transferable skills, such as communication, collaboration, and problem‐solving, was deemed essential to prepare students for the complexities of the 21st‐century workforce. The conference explored innovative approaches to integrating these skills into the science curriculum, emphasizing the importance of self‐reflection and mentorship in fostering their development.

Using arts to nurture reflective attitudes and the importance of interdisciplinary approaches in education were highlighted. Encouraging learners to engage with art as a means of self‐discovery and critical thinking and to reflect on their experiences can lead to deeper learning and personal growth.

The professional development of all those who teach was recognized as a cornerstone of educational excellence. The conference emphasized the need for continuous learning and growth among educators, fostering a culture of reflection, innovation, and collaboration. The National Professional Development Framework of Ireland, which supports all who teach in higher education by providing a structured outline of activities aligned with teaching and learning goals was emphasized. Launched in 2016, this framework aims to empower staff to create, discover, and engage in meaningful personal and professional development, to engage in peer dialog and support and to enhance and develop the pedagogy of individual disciplines and to enable learning from other disciplines.

**Fig. 4:**
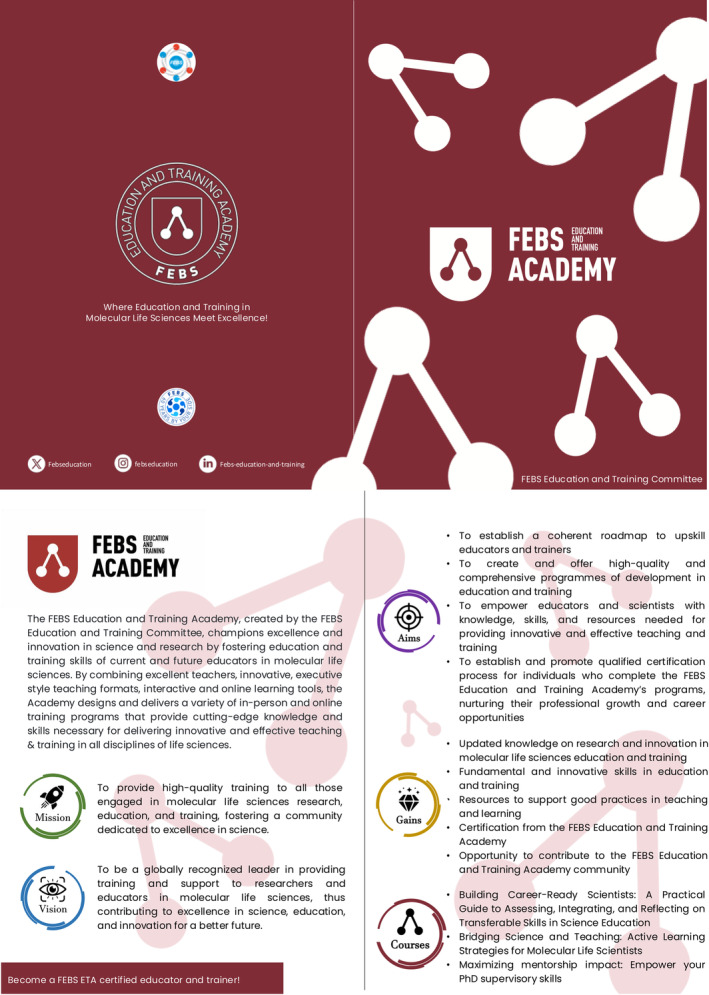
FEBS Education and Training Academy Overview. This infographic presents key information about the FEBS Education and Training Academy, an initiative of the FEBS Education and Training Committee. It outlines the Academy's mission to foster excellence in molecular life sciences education, its vision for global leadership in training, and its strategic objectives. The figure highlights the benefits for participants, including updated knowledge, innovative skills, resources, and certification opportunities. It also showcases sample training programs offered by the Academy, demonstrating its commitment to enhancing education and training in molecular life sciences across Europe.

The importance of mentorship and peer support was highlighted, along with the value of structured professional development programs that cater to the diverse needs and aspirations of educators. The FEBS Education and Training Academy, launched during the conference, exemplifies this commitment to empowering educators through high‐quality training and fostering a community dedicated to excellence in science education (Fig. 4).

The need for tailored approaches to enhance disciplinary excellence was also underscored, with The Disciplinary Excellence in Teaching, Learning, and Assessment (DELTA) Framework in Ireland serving as a model for a strategic path for disciplines to enhance teaching. The framework is recognized through the DELTA National Award, which aims to support staff across disciplines to work collaboratively to articulate, evidence, and plan their engagement in and commitment to teaching and learning enhancement, toward student success. Such a model provides a capacity building and planning tool for forward‐looking discipline groups, enabling the sharing of good practices for enhancing teaching and learning within and across disciplines.

## From recommendations to action: A roadmap for molecular life sciences education

**Fig. 5:**
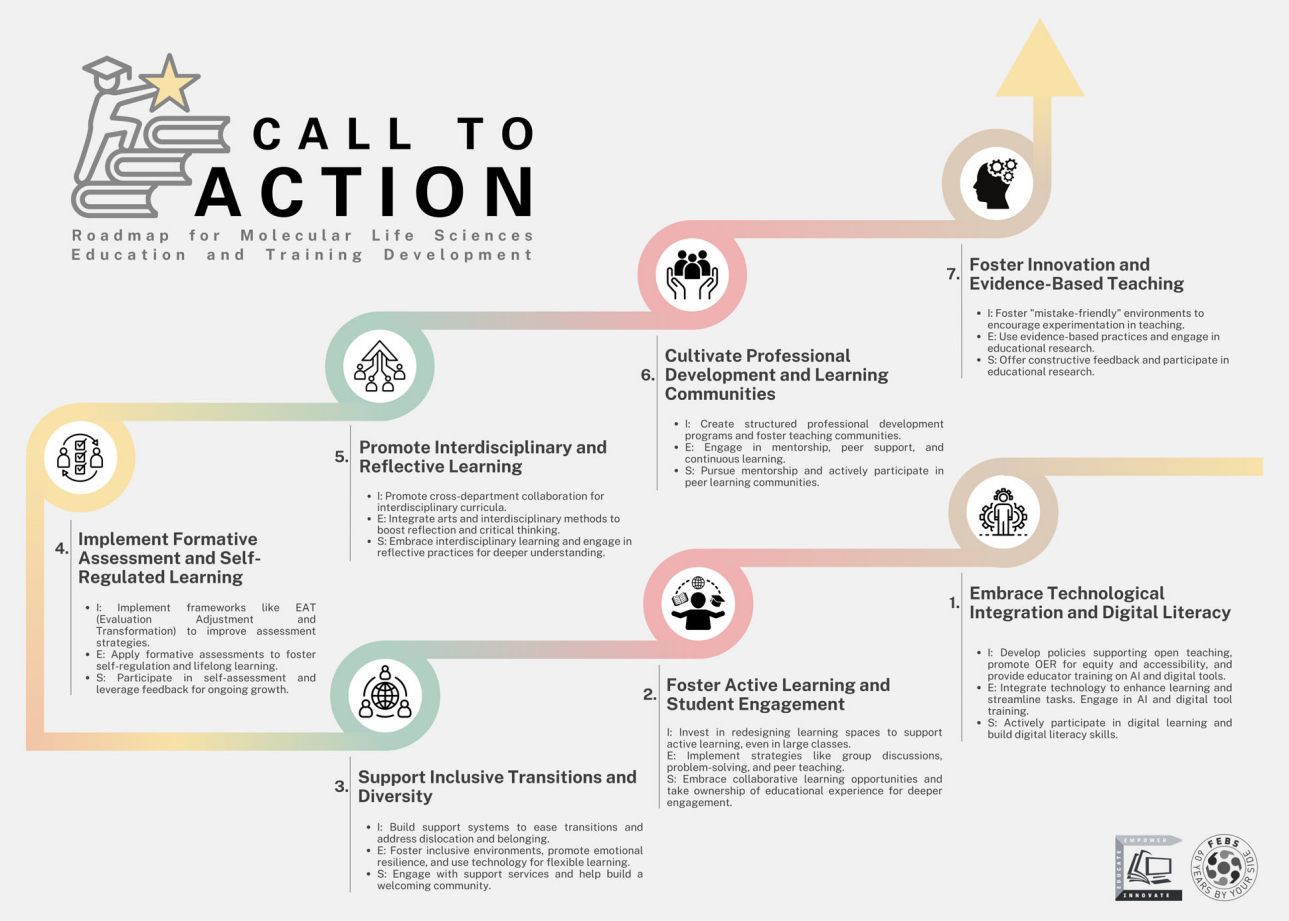
Call to Action—Roadmap for Molecular Life Sciences Education. This infographic outlines seven key strategic areas for advancing education in molecular life sciences, as identified during the FEBS Education & Training Conference 2024. Each segment presents actionable recommendations for institutions (I), educators (E), and students (S), covering: (1) Technological Integration and Digital Literacy, (2) Active Learning and Student Engagement, (3) Inclusive Transitions and Diversity, (4) Formative Assessment and Self‐Regulated Learning, (5) Interdisciplinary and Reflective Learning, (6) Professional Development and Learning Communities, and (7) Innovation and Evidence‐Based Teaching. This roadmap provides a comprehensive guide for stakeholders to collaboratively enhance the quality and effectiveness of molecular life sciences education.

The insights gained from the FEBS Education and Training Conference highlight the need for transformative changes in molecular life sciences education. To address the identified challenges and opportunities identified, we propose the following actionable strategies that combine our recommendations with a call to action for all stakeholders in the field (Fig. 5):

### Embrace technological integration and digital literacy

Actions for Institutions:
  ○Develop and implement policies that support digital and open teaching and learning, including the promotion of Open Educational Resources (OER) to ensure equity and accessibility in education, allowing resources to be freely accessible, shared, and adapted.
  ○Provide training for educators on the effective use of AI and digital tools to enhance teaching and learning experiences.
  ○Recognize teaching excellence with similar esteem to research excellence, in career evaluations.
Actions for Educators:
  ○Thoughtfully integrate technology as a powerful tool to enhance learning experiences and to streamline administrative tasks.
  ○Engage in training programs to understand how to embed active learning in teaching, and how to effectively use AI and digital tools to enhance teaching and learning experiences.
Actions for Students: Actively participate in digital learning opportunities and develop critical digital literacy skills.

### Foster active learning and student engagement

- Action for Institutions: Invest in redesigning learning spaces to facilitate active learning strategies, even with large classes.
- Action for Educators: Embed diverse active learning techniques such as group discussions, problem‐solving activities, and peer teaching.
- Action for Students: Embrace opportunities for collaborative learning and take ownership of the personal educational journey.

### Support inclusive transitions and diversity

Action for Institutions: Establish comprehensive support systems to ease the transition from school to university, addressing challenges like dislocation and loss of a sense of belonging.
Action for Educators:
  ○Create inclusive learning environments that cater to diverse student needs and promote emotional resilience.
  ○Utilize technology to offer flexible and asynchronous learning opportunities that promote self‐regulated learning and adaptability.
Action for Students: Engage with support services and contribute to creating a welcoming community for all learners.

### Implement formative assessment and self‐regulated learning

- Action for Institutions: Adopt frameworks like the EAT framework to guide assessment practices.
- Action for Educators: Develop assessment literacy and integrate formative assessment techniques to enhance self‐regulated learning and lifelong learning competencies.
- Action for Students: Actively engage in self‐assessment and use feedback to drive continuous improvement.

### Promote interdisciplinary and reflective learning

- Action for Institutions: Encourage cross‐departmental collaboration to develop interdisciplinary curricula.
- Action for Educators: Integrate arts and interdisciplinary approaches in the curriculum to foster reflective attitudes and critical thinking and to promote a sense of belonging.
- Action for Students: Embrace opportunities for interdisciplinary learning and engage in reflective practices to deepen your understanding.

### Cultivate professional development and learning communities

- Action for Institutions: Establish structured professional development programs that cater to the diverse needs of educators and foster the creation of communities of practice for sharing practices of teaching and learning.
- Action for Educators: Actively participate in mentorship programs, peer support networks, communities of practice, and other continuous learning opportunities.
- Action for Students: Seek mentorship opportunities and contribute to peer learning communities.

### Foster innovation and evidence‐based teaching

- Action for Institutions: Create ‘mistake‐friendly’ environments that encourage experimentation and innovation in teaching methods; cultivate and celebrate excellence in teaching.
- Action for Educators: Adopt evidence‐based teaching practices and consider contributing to educational research in molecular life sciences.
- Action for Students: Provide constructive feedback on teaching methods and participate in educational research studies.

We call upon all stakeholders—policymakers, institutional leaders, educators, and students—to embrace these strategies and to take concrete steps toward their implementation. Together, we can create a dynamic, inclusive, and forward‐thinking educational ecosystem that prepares molecular life scientists to shape the future of our field and to contribute meaningfully to society. This concerted effort will ensure that the next generation of scientists is equipped with the knowledge, skills, and values necessary to tackle complex global challenges and to drive innovation in the field.

## Conclusion

The Molecular Life Sciences Education Conference highlighted the urgent need for innovation and collaboration in education. This document serves as a guiding framework for future actions, emphasizing the importance of education, and training in shaping the next generation of scientists. The insights, recommendations, and collaborative spirit that emerged from the conference can undoubtedly shape the future of education, ensuring that the next generation of scientists is equipped with the knowledge, skills, and values to tackle the complex challenges that lie ahead.

As we move forward, it is imperative that we continue to build upon the momentum generated by the FEBS ETC. By embracing innovation, fostering collaboration, and prioritizing the needs of our students, we can create a brighter future for molecular life sciences education, one that empowers learners to become agents of change in a world that desperately needs their expertise and passion. By working together, we can overcome challenges and ensure a bright future for molecular life sciences education.

## Conflict of interest

The authors declare no conflict of interest.

## Author contributions

LV: participated in writing the manuscript, providing initial material; NS: participated in writing the manuscript, design of illustrative material; LVM: participated in writing and correcting the manuscript; MJC: participated in writing and correcting the manuscript; DP: participated in writing and correcting the manuscript; FM: participated in writing the manuscript, providing initial material; JD: participated in writing the manuscript; FGS: participated in writing the manuscript, preparing overall structure of the manuscript.

